# {3,3′-Bis[(anthracen-9-yl)meth­yl]-1,1′-[(ethane-1,2-diyldi­oxy)bis­(ethane-1,2-di­yl)]bis­(imidazol-2-yl­idene)}mercury(II) bis­(hexa­fluoridophosphate) acetonitrile disolvate

**DOI:** 10.1107/S1600536812005958

**Published:** 2012-02-17

**Authors:** Jun-Wen Wang, Yue Guo, Gui-Ying Dong, Yu Gu, Di-Si Bai

**Affiliations:** a College of Chemical and Materials Science, Shanxi Normal University, Linfen 041004, People’s Republic of China; b College of Chemical Engineering, Hebei United University, Tangshan 063009, People’s Republic of China; c Qian’an College, Hebei United University, Tangshan 063009, People’s Republic of China

## Abstract

In the title compound, [Hg(C_42_H_38_N_4_O_2_)](PF_6_)_2_·2CH_3_CN, the Hg^II^ cation lies on a twofold axis which is also the inter­nal symmetry element of the complete cationic complex. The Hg^II^ cation is coordinated by two symmetry-related C(carbene) atoms [Hg—C = 2.058 (9) Å] in a nearly linear geometry, with a C—Hg—C angle of 175.8 (5)°. There are weak inter­molecular C—H⋯F inter­actions in the crystal packing between an F atom of a hexa­fluoridophosphate anion and a –CH_2_– group of the bis-N-heterocyclic carbene ligand.

## Related literature

For related bis-*N*-heterocyclic carbene structures, see: Arduengo *et al.* (1991 ▶); Nielsen *et al.* (2006 ▶); Guo & Dong (2009 ▶).
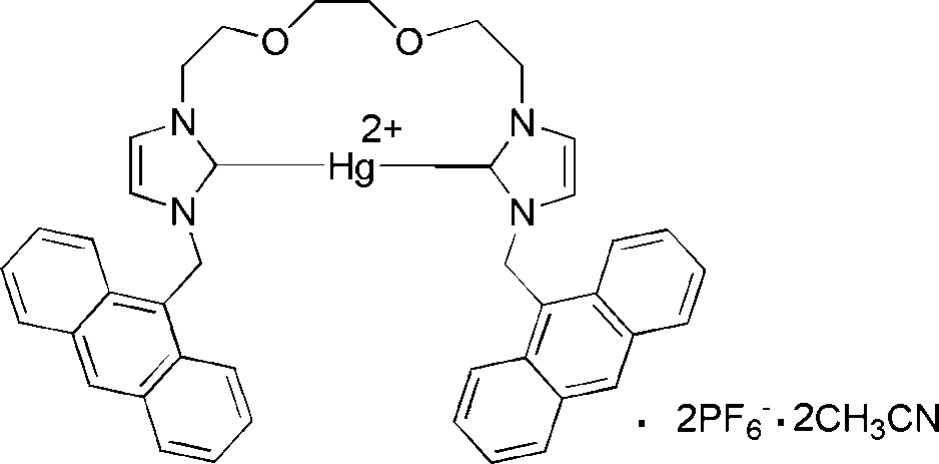


## Experimental

### 

#### Crystal data

[Hg(C_42_H_38_N_4_O_2_)](PF_6_)_2_·2C_2_H_3_N
*M* *_r_* = 1203.4
Orthorhombic, 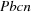

*a* = 19.774 (5) Å
*b* = 9.774 (3) Å
*c* = 24.250 (6) Å
*V* = 4687 (2) Å^3^
*Z* = 4
Mo *K*α radiation
μ = 3.45 mm^−1^
*T* = 298 K
0.24 × 0.08 × 0.06 mm

#### Data collection

Bruker APEXII CCD area-detector diffractometer
Absorption correction: multi-scan *SADABS* (Sheldrick, 1996 ▶) *T*_min_ = 0.775, *T*_max_ = 0.864
25351 measured reflections
4804 independent reflections
3011 reflections with *I* > 2σ(*I*)
*R*_int_ = 0.086

#### Refinement

*R*[*F* ^2^ > 2σ(*F* ^2^)] = 0.065
*wR*(*F* ^2^) = 0.169
*S* = 1.10
4804 reflections
313 parameters
3 restraints
H-atom parameters constrained
Δρ_max_ = 1.46 e Å^−3^
Δρ_min_ = −1.80 e Å^−3^

### 

Data collection: *APEX2* (Bruker, 2007 ▶); cell refinement: *SAINT* (Bruker, 2007 ▶); data reduction: *SAINT*; program(s) used to solve structure: *SHELXS97* (Sheldrick, 2008 ▶); program(s) used to refine structure: *SHELXL97* (Sheldrick, 2008 ▶); molecular graphics: *SHELXTL* (Sheldrick, 2008 ▶); software used to prepare material for publication: *SHELXTL*.

## Supplementary Material

Crystal structure: contains datablock(s) I, global. DOI: 10.1107/S1600536812005958/vn2031sup1.cif

Structure factors: contains datablock(s) I. DOI: 10.1107/S1600536812005958/vn2031Isup2.hkl

Additional supplementary materials:  crystallographic information; 3D view; checkCIF report

## Figures and Tables

**Table 1:** Selected bond lengths (Å)

Hg1—C1	2.058 (9)
P1—F5	1.561 (8)

**Table 2:** Hydrogen-bond geometry (Å, °)

*D*—H⋯*A*	*D*—H	H⋯*A*	*D*⋯*A*	*D*—H⋯*A*
C4—H4*B*⋯F5	0.97	2.48	3.265 (13)	137

**Fig. 1.**
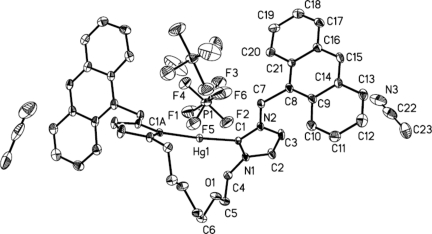
The molecular structure of the title compound showing atomic displacement ellipsoids at the 30% probability level [symmetry code A: -x, y, 0.5 - z]. H atoms are omitted for clarity.

## supplementary crystallographic information

### Comment

N-heterocyclic carbene (NHC) ligands derived from imidazolium salts have seen an increasing use in organometallic chemistry and homogeneous catalysis (Arduengo *et al.*,1991). The mercury and silver complexes of bis-NHC ligands bearing a weakly coordinating ether functionality have been reported before (Guo & Dong, 2009; Nielsen *et al.*, 2006). To study further the coordination chemistry of this kind of ligands, we report here the crystal structure of the title complex, (I)

The asymmetric unit of the title compound [C_42_H_38_HgN_4_O_2_]^2+^.2(PF_6_)^-^.2CH_3_CN (I) consists of one half of the [3,3'-Bis(9-anthracenylmethyl)-1,1'-(2,2'-oxydiethylene)bis-(imidazol- 2-ylidene)]mercury(II) cation, one hexafluorophosphate anion and one acetonitrile solvate molecule. The complete complex (Fig. 1) is generated by a crystallographic two-fold axis on which the Hg^II^ cation is situated. The Hg^II^ cation of (I) is coordinated by an anthracenyl-carbene ligand adopting a *cis*-conformation, the geometry of the Hg^II^ coordination being nearly linear, formed by two symmetry related C(carbene) atoms [Hg—C = 2.058 (9) Å, C— Hg—C = 175.8 (5)°]. The crystal packing exhibits intermolecular C—H···F weak interaction between the organic C atoms and the F atom of hexafluorophosphate anion with H4B···F distance of 2.48 Å.

### Experimental

[3,3'-Bis(9-anthracenylmethyl)-1,1'-(2,2'- oxydiethylene)bis-imidazolium] hexafluorophosphate salt (522 mg, 0.566 mmol) was mixed with anhydrous Hg(OAc)_2_ (181 mg, 0.566 mmol) in acetonitrile (100 ml) (under argon) and heated under reflux for 2 d and then cooled to room temperature. The acetonitrile was removed *in vacuo* to yield a white solid which was washed with methanol to give the crude product. Colourless single crystals of the title compound were obtained by recrystallization from acetonitile and ethyl ether (yield: 75.3%).

### Refinement

The acetonitrile solvant molecule shows slight positional disorder. Instead of treating the disorder with split sites, the geometry of the molecule was rather regularized with the following three distance restraints: N3—C23: 2.60 (1) Å, N3—C22: 1.10 (1) Å and C22—C23: 1.50 (1) Å. The aromatic [C—H = 0.93 Å and *U*_iso_(H) = 1.2*U*_eq_(C)] and methylene H atoms [C—H = 0.96 Å and *U*_iso_(H) = 1.5*U*_eq_(C)] were included in the refinement using a riding-model approximation.

### Crystal data

**Table:**

[Hg(C_42_H_38_N_4_O_2_)](PF_6_)_2_·2C_2_H_3_N	*F*(000) = 2384
*M**_r_* = 1203.4	*D*_x_ = 1.705 Mg m^−^^3^
Orthorhombic, *P**b**c**n*	Mo *K*α radiation, λ = 0.71073 Å
Hall symbol: -P 2n 2ab	Cell parameters from 990 reflections
*a* = 19.774 (5) Å	θ = 2.7–24.2°
*b* = 9.774 (3) Å	µ = 3.45 mm^−^^1^
*c* = 24.250 (6) Å	*T* = 298 K
*V* = 4687 (2) Å^3^	Block, colourless
*Z* = 4	0.24 × 0.08 × 0.06 mm

### Data collection

**Table:**

Bruker APEXII CCD area-detector diffractometer	4804 independent reflections
Radiation source: fine-focus sealed tube	3011 reflections with *I* > 2σ(*I*)
Graphite monochromator	*R*_int_ = 0.086
φ and ω scans	θ_max_ = 26.4°, θ_min_ = 2.3°
Absorption correction: multi-scan *SADABS* (Sheldrick, 1996)	*h* = −24→21
*T*_min_ = 0.775, *T*_max_ = 0.864	*k* = −8→12
25351 measured reflections	*l* = −30→29

### Refinement

**Table:**

Refinement on *F*^2^	Primary atom site location: structure-invariant direct methods
Least-squares matrix: full	Secondary atom site location: difference Fourier map
*R*[*F*^2^ > 2σ(*F*^2^)] = 0.065	Hydrogen site location: inferred from neighbouring sites
*wR*(*F*^2^) = 0.169	H-atom parameters constrained
*S* = 1.10	*w* = 1/[σ^2^(*F*_o_^2^) + (0.0885*P*)^2^] where *P* = (*F*_o_^2^ + 2*F*_c_^2^)/3
4804 reflections	(Δ/σ)_max_ = 0.001
313 parameters	Δρ_max_ = 1.46 e Å^−^^3^
3 restraints	Δρ_min_ = −1.80 e Å^−^^3^

### Special details

**Table:**

Geometry. All e.s.d.'s (except the e.s.d. in the dihedral angle between two l.s. planes) are estimated using the full covariance matrix. The cell e.s.d.'s are taken into account individually in the estimation of e.s.d.'s in distances, angles and torsion angles; correlations between e.s.d.'s in cell parameters are only used when they are defined by crystal symmetry. An approximate (isotropic) treatment of cell e.s.d.'s is used for estimating e.s.d.'s involving l.s. planes.
Refinement. Refinement of *F*^2^ against ALL reflections. The weighted *R*-factor *wR* and goodness of fit *S* are based on *F*^2^, conventional *R*-factors *R* are based on *F*, with *F* set to zero for negative *F*^2^. The threshold expression of *F*^2^ > σ(*F*^2^) is used only for calculating *R*-factors(gt) *etc*. and is not relevant to the choice of reflections for refinement. *R*-factors based on *F*^2^ are statistically about twice as large as those based on *F*, and *R*-factors based on ALL data will be even larger.

### Fractional atomic coordinates and isotropic or equivalent isotropic displacement parameters (Å^2^)

**Table:**

	*x*	*y*	*z*	*U*_iso_*/*U*_eq_	
Hg1	0.0000	0.46027 (4)	0.2500	0.03456 (17)	
N1	0.1519 (4)	0.5080 (8)	0.2447 (3)	0.0373 (17)	
N2	0.1200 (3)	0.3926 (8)	0.1754 (3)	0.0378 (17)	
O1	0.0679 (4)	0.7289 (7)	0.2621 (3)	0.054 (2)	
C1	0.0977 (4)	0.4525 (8)	0.2209 (3)	0.0316 (18)	
C2	0.2094 (5)	0.4854 (12)	0.2147 (5)	0.057 (3)	
H2	0.2531	0.5141	0.2229	0.068*	
C3	0.1889 (5)	0.4122 (11)	0.1705 (4)	0.052 (3)	
H3	0.2162	0.3808	0.1419	0.062*	
C4	0.1511 (5)	0.5826 (10)	0.2972 (4)	0.044 (2)	
H4A	0.1957	0.5791	0.3136	0.053*	
H4B	0.1198	0.5379	0.3222	0.053*	
C5	0.1304 (5)	0.7289 (10)	0.2901 (4)	0.049 (3)	
H5A	0.1258	0.7729	0.3257	0.058*	
H5B	0.1641	0.7780	0.2688	0.058*	
C6	0.0285 (6)	0.8505 (10)	0.2693 (4)	0.048 (2)	
H6A	0.0565	0.9303	0.2628	0.057*	
H6B	0.0117	0.8548	0.3068	0.057*	
C7	0.0762 (5)	0.3126 (10)	0.1375 (4)	0.046 (2)	
H7A	0.0425	0.3731	0.1218	0.055*	
H7B	0.0527	0.2429	0.1586	0.055*	
C8	0.1157 (5)	0.2431 (11)	0.0902 (4)	0.046 (2)	
C9	0.1315 (5)	0.3178 (11)	0.0428 (4)	0.048 (3)	
C10	0.1182 (6)	0.4599 (12)	0.0370 (5)	0.060 (3)	
H10	0.0982	0.5063	0.0663	0.073*	
C11	0.1334 (8)	0.5304 (11)	−0.0093 (5)	0.067 (4)	
H11	0.1218	0.6223	−0.0124	0.081*	
C12	0.1673 (6)	0.4629 (14)	−0.0533 (5)	0.070 (4)	
H12	0.1793	0.5117	−0.0847	0.084*	
C13	0.1823 (5)	0.3289 (13)	−0.0497 (4)	0.058 (3)	
H13	0.2038	0.2861	−0.0791	0.069*	
C14	0.1654 (5)	0.2502 (11)	−0.0012 (4)	0.050 (3)	
C15	0.1800 (5)	0.1125 (12)	0.0019 (4)	0.050 (3)	
H15	0.2000	0.0694	−0.0282	0.061*	
C16	0.1655 (5)	0.0367 (11)	0.0488 (4)	0.048 (3)	
C17	0.1790 (5)	−0.1056 (12)	0.0516 (5)	0.055 (3)	
H17	0.1975	−0.1491	0.0210	0.066*	
C18	0.1657 (5)	−0.1800 (12)	0.0975 (5)	0.061 (3)	
H18	0.1764	−0.2725	0.0985	0.073*	
C19	0.1358 (6)	−0.1179 (11)	0.1431 (5)	0.064 (3)	
H19	0.1259	−0.1703	0.1741	0.076*	
C20	0.1209 (6)	0.0192 (11)	0.1432 (5)	0.054 (3)	
H20	0.1028	0.0592	0.1747	0.065*	
C21	0.1330 (5)	0.1013 (10)	0.0953 (4)	0.041 (2)	
N3	0.5038 (11)	0.1282 (19)	0.1170 (10)	0.146 (8)	
C22	0.5063 (9)	0.217 (2)	0.0890 (8)	0.117 (9)	
C23	0.5048 (8)	0.334 (2)	0.0493 (9)	0.145 (9)	
H23A	0.4653	0.3277	0.0265	0.217*	
H23B	0.5445	0.3318	0.0266	0.217*	
H23C	0.5037	0.4190	0.0694	0.217*	
P1	0.13116 (17)	0.1670 (3)	0.34660 (15)	0.0625 (8)	
F1	0.1456 (7)	0.1821 (11)	0.4086 (4)	0.165 (5)	
F2	0.1925 (4)	0.2599 (8)	0.3349 (5)	0.136 (4)	
F3	0.1723 (6)	0.0382 (8)	0.3418 (7)	0.194 (7)	
F4	0.0695 (5)	0.0783 (9)	0.3592 (4)	0.128 (4)	
F5	0.0880 (4)	0.3006 (8)	0.3479 (5)	0.125 (3)	
F6	0.1159 (9)	0.1570 (13)	0.2854 (4)	0.219 (8)	

### Atomic displacement parameters (Å^2^)

**Table:**

	*U*^11^	*U*^22^	*U*^33^	*U*^12^	*U*^13^	*U*^23^
Hg1	0.0341 (3)	0.0382 (3)	0.0314 (2)	0.000	0.0074 (3)	0.000
N1	0.040 (4)	0.047 (4)	0.025 (4)	−0.003 (3)	0.002 (4)	−0.010 (4)
N2	0.024 (4)	0.049 (4)	0.041 (4)	0.001 (4)	0.006 (3)	−0.007 (4)
O1	0.067 (5)	0.037 (3)	0.057 (5)	−0.002 (3)	−0.013 (3)	−0.017 (3)
C1	0.039 (5)	0.034 (4)	0.022 (4)	−0.002 (4)	0.006 (4)	0.002 (4)
C2	0.029 (5)	0.081 (8)	0.061 (7)	−0.009 (5)	0.007 (5)	−0.032 (6)
C3	0.032 (5)	0.076 (7)	0.047 (6)	0.001 (5)	0.013 (5)	−0.011 (6)
C4	0.040 (6)	0.047 (5)	0.045 (6)	−0.002 (5)	−0.004 (5)	−0.004 (5)
C5	0.054 (6)	0.047 (6)	0.045 (6)	−0.009 (5)	−0.013 (5)	−0.015 (5)
C6	0.064 (6)	0.036 (5)	0.043 (5)	0.001 (5)	−0.007 (5)	−0.002 (4)
C7	0.040 (5)	0.052 (6)	0.046 (6)	−0.007 (5)	0.013 (5)	−0.013 (5)
C8	0.041 (5)	0.060 (6)	0.036 (5)	−0.010 (5)	0.005 (4)	−0.018 (5)
C9	0.037 (5)	0.060 (6)	0.048 (6)	−0.009 (5)	0.006 (5)	−0.024 (5)
C10	0.067 (8)	0.065 (7)	0.050 (7)	−0.010 (6)	0.008 (6)	−0.016 (6)
C11	0.087 (10)	0.062 (8)	0.053 (8)	−0.006 (7)	−0.002 (6)	0.005 (6)
C12	0.060 (8)	0.096 (11)	0.055 (7)	−0.007 (8)	−0.008 (6)	0.001 (7)
C13	0.061 (7)	0.077 (8)	0.035 (5)	−0.006 (7)	0.002 (5)	−0.008 (6)
C14	0.046 (6)	0.061 (6)	0.042 (6)	−0.013 (6)	0.008 (5)	−0.012 (5)
C15	0.047 (6)	0.064 (6)	0.040 (6)	0.000 (6)	0.011 (5)	−0.014 (5)
C16	0.034 (5)	0.061 (7)	0.047 (6)	−0.005 (5)	−0.003 (4)	−0.020 (5)
C17	0.040 (6)	0.061 (7)	0.065 (7)	0.000 (5)	−0.004 (5)	−0.025 (6)
C18	0.053 (7)	0.057 (7)	0.073 (8)	0.001 (6)	−0.016 (6)	−0.004 (6)
C19	0.079 (8)	0.047 (6)	0.066 (7)	−0.011 (6)	−0.009 (7)	0.000 (6)
C20	0.055 (7)	0.064 (7)	0.045 (6)	−0.020 (6)	−0.001 (5)	−0.008 (5)
C21	0.034 (5)	0.050 (5)	0.040 (5)	−0.011 (5)	0.001 (4)	−0.012 (5)
N3	0.114 (13)	0.110 (13)	0.21 (2)	0.013 (13)	−0.025 (13)	−0.071 (16)
C22	0.044 (9)	0.124 (17)	0.18 (3)	0.015 (15)	−0.002 (14)	−0.085 (18)
C23	0.097 (14)	0.16 (2)	0.18 (2)	0.033 (15)	0.064 (13)	−0.022 (18)
P1	0.068 (2)	0.0389 (14)	0.080 (2)	−0.0070 (15)	−0.0066 (18)	−0.0066 (15)
F1	0.258 (14)	0.134 (8)	0.103 (7)	−0.033 (9)	−0.075 (8)	−0.014 (7)
F2	0.091 (6)	0.088 (6)	0.228 (12)	−0.023 (5)	0.030 (7)	0.004 (7)
F3	0.179 (11)	0.056 (5)	0.35 (2)	0.026 (6)	0.102 (12)	−0.006 (8)
F4	0.108 (7)	0.121 (7)	0.155 (9)	−0.050 (6)	0.032 (6)	−0.023 (7)
F5	0.078 (5)	0.089 (6)	0.208 (11)	0.011 (5)	0.000 (6)	0.024 (7)
F6	0.42 (2)	0.158 (11)	0.084 (7)	−0.145 (14)	−0.030 (10)	−0.001 (7)

### Geometric parameters (Å, º)

**Table:**

Hg1—C1	2.058 (9)	C11—C12	1.423 (17)
Hg1—C1^i^	2.058 (9)	C11—H11	0.9300
N1—C1	1.334 (11)	C12—C13	1.345 (15)
N1—C2	1.367 (12)	C12—H12	0.9300
N1—C4	1.466 (11)	C13—C14	1.446 (14)
N2—C1	1.324 (10)	C13—H13	0.9300
N2—C3	1.380 (11)	C14—C15	1.378 (14)
N2—C7	1.485 (11)	C15—C16	1.388 (14)
O1—C5	1.410 (11)	C15—H15	0.9300
O1—C6	1.432 (12)	C16—C17	1.417 (15)
C2—C3	1.351 (14)	C16—C21	1.444 (13)
C2—H2	0.9300	C17—C18	1.355 (15)
C3—H3	0.9300	C17—H17	0.9300
C4—C5	1.497 (13)	C18—C19	1.393 (15)
C4—H4A	0.9700	C18—H18	0.9300
C4—H4B	0.9700	C19—C20	1.371 (15)
C5—H5A	0.9700	C19—H19	0.9300
C5—H5B	0.9700	C20—C21	1.431 (14)
C6—C6^i^	1.46 (2)	C20—H20	0.9300
C6—H6A	0.9700	N3—C22	1.104 (8)
C6—H6B	0.9700	C22—C23	1.497 (8)
C7—C8	1.546 (12)	C23—H23A	0.9600
C7—H7A	0.9700	C23—H23B	0.9600
C7—H7B	0.9700	C23—H23C	0.9600
C8—C9	1.397 (14)	P1—F6	1.518 (11)
C8—C21	1.433 (14)	P1—F3	1.503 (9)
C9—C10	1.421 (14)	P1—F4	1.527 (8)
C9—C14	1.422 (13)	P1—F1	1.539 (10)
C10—C11	1.352 (16)	P1—F2	1.540 (8)
C10—H10	0.9300	P1—F5	1.561 (8)
			
C1—Hg1—C1^i^	175.8 (5)	C13—C12—C11	120.5 (12)
C1—N1—C2	111.9 (7)	C13—C12—H12	119.8
C1—N1—C4	124.6 (7)	C11—C12—H12	119.8
C2—N1—C4	123.5 (8)	C12—C13—C14	121.4 (11)
C1—N2—C3	109.9 (8)	C12—C13—H13	119.3
C1—N2—C7	123.6 (7)	C14—C13—H13	119.3
C3—N2—C7	126.5 (7)	C15—C14—C9	120.8 (10)
C5—O1—C6	114.7 (7)	C15—C14—C13	121.0 (10)
N2—C1—N1	105.8 (8)	C9—C14—C13	118.1 (10)
N2—C1—Hg1	128.0 (7)	C14—C15—C16	121.5 (9)
N1—C1—Hg1	126.3 (6)	C14—C15—H15	119.3
C3—C2—N1	105.0 (9)	C16—C15—H15	119.3
C3—C2—H2	127.5	C15—C16—C17	121.6 (10)
N1—C2—H2	127.5	C15—C16—C21	119.9 (10)
C2—C3—N2	107.5 (8)	C17—C16—C21	118.4 (10)
C2—C3—H3	126.3	C18—C17—C16	121.9 (10)
N2—C3—H3	126.3	C18—C17—H17	119.0
N1—C4—C5	112.2 (8)	C16—C17—H17	119.0
N1—C4—H4A	109.2	C17—C18—C19	120.1 (11)
C5—C4—H4A	109.2	C17—C18—H18	120.0
N1—C4—H4B	109.2	C19—C18—H18	120.0
C5—C4—H4B	109.2	C20—C19—C18	121.1 (11)
H4A—C4—H4B	107.9	C20—C19—H19	119.4
O1—C5—C4	107.1 (8)	C18—C19—H19	119.4
O1—C5—H5A	110.3	C19—C20—C21	120.8 (10)
C4—C5—H5A	110.3	C19—C20—H20	119.6
O1—C5—H5B	110.3	C21—C20—H20	119.6
C4—C5—H5B	110.3	C20—C21—C8	125.0 (9)
H5A—C5—H5B	108.5	C20—C21—C16	117.5 (10)
O1—C6—C6^i^	110.0 (7)	C8—C21—C16	117.5 (9)
O1—C6—H6A	109.7	N3—C22—C23	176 (3)
C6^i^—C6—H6A	109.7	C22—C23—H23A	109.5
O1—C6—H6B	109.7	C22—C23—H23B	109.5
C6^i^—C6—H6B	109.7	H23A—C23—H23B	109.5
H6A—C6—H6B	108.2	C22—C23—H23C	109.5
N2—C7—C8	113.3 (7)	H23A—C23—H23C	109.5
N2—C7—H7A	108.9	H23B—C23—H23C	109.5
C8—C7—H7A	108.9	F6—P1—F3	88.8 (9)
N2—C7—H7B	108.9	F6—P1—F4	90.0 (7)
C8—C7—H7B	108.9	F3—P1—F4	88.4 (6)
H7A—C7—H7B	107.7	F6—P1—F1	178.0 (8)
C9—C8—C21	121.6 (9)	F3—P1—F1	93.2 (8)
C9—C8—C7	119.6 (9)	F4—P1—F1	90.4 (6)
C21—C8—C7	118.7 (9)	F6—P1—F2	90.9 (7)
C8—C9—C10	123.4 (9)	F3—P1—F2	93.1 (5)
C8—C9—C14	118.6 (10)	F4—P1—F2	178.3 (6)
C10—C9—C14	117.9 (10)	F1—P1—F2	88.7 (7)
C11—C10—C9	122.6 (11)	F6—P1—F5	88.0 (8)
C11—C10—H10	118.7	F3—P1—F5	176.8 (9)
C9—C10—H10	118.7	F4—P1—F5	92.0 (5)
C10—C11—C12	119.4 (11)	F1—P1—F5	90.0 (6)
C10—C11—H11	120.3	F2—P1—F5	86.6 (5)
C12—C11—H11	120.3		
			
C3—N2—C1—N1	−0.7 (10)	C9—C10—C11—C12	−3.4 (18)
C7—N2—C1—N1	177.2 (8)	C10—C11—C12—C13	2.1 (19)
C3—N2—C1—Hg1	179.4 (7)	C11—C12—C13—C14	−1.0 (18)
C7—N2—C1—Hg1	−2.7 (13)	C8—C9—C14—C15	2.8 (15)
C2—N1—C1—N2	0.5 (11)	C10—C9—C14—C15	179.9 (10)
C4—N1—C1—N2	−178.5 (8)	C8—C9—C14—C13	−179.2 (9)
C2—N1—C1—Hg1	−179.6 (8)	C10—C9—C14—C13	−2.1 (14)
C4—N1—C1—Hg1	1.4 (13)	C12—C13—C14—C15	179.0 (11)
C1—N1—C2—C3	−0.1 (13)	C12—C13—C14—C9	1.0 (16)
C4—N1—C2—C3	178.9 (9)	C9—C14—C15—C16	−3.6 (16)
N1—C2—C3—N2	−0.3 (13)	C13—C14—C15—C16	178.5 (10)
C1—N2—C3—C2	0.6 (12)	C14—C15—C16—C17	178.5 (10)
C7—N2—C3—C2	−177.2 (10)	C14—C15—C16—C21	1.6 (15)
C1—N1—C4—C5	−82.8 (11)	C15—C16—C17—C18	179.3 (10)
C2—N1—C4—C5	98.3 (11)	C21—C16—C17—C18	−3.7 (15)
C6—O1—C5—C4	157.9 (9)	C16—C17—C18—C19	2.1 (16)
N1—C4—C5—O1	54.2 (11)	C17—C18—C19—C20	−1.6 (17)
C5—O1—C6—C6^i^	169.4 (10)	C18—C19—C20—C21	2.7 (16)
C1—N2—C7—C8	−175.1 (8)	C19—C20—C21—C8	176.4 (10)
C3—N2—C7—C8	2.4 (14)	C19—C20—C21—C16	−4.3 (14)
N2—C7—C8—C9	−83.4 (11)	C9—C8—C21—C20	177.5 (9)
N2—C7—C8—C21	99.1 (10)	C7—C8—C21—C20	−5.0 (14)
C21—C8—C9—C10	−177.0 (10)	C9—C8—C21—C16	−1.8 (14)
C7—C8—C9—C10	5.6 (15)	C7—C8—C21—C16	175.7 (8)
C21—C8—C9—C14	0.0 (14)	C15—C16—C21—C20	−178.3 (9)
C7—C8—C9—C14	−177.5 (8)	C17—C16—C21—C20	4.7 (13)
C8—C9—C10—C11	−179.6 (11)	C15—C16—C21—C8	1.1 (13)
C14—C9—C10—C11	3.4 (17)	C17—C16—C21—C8	−175.9 (8)

Symmetry code: (i) −*x*, *y*, −*z*+1/2.

### Hydrogen-bond geometry (Å, º)

**Table:**

*D*—H···*A*	*D*—H	H···*A*	*D*···*A*	*D*—H···*A*
C4—H4*B*···F5	0.97	2.48	3.265 (13)	137
